# The Guild of St. Luke

**Published:** 1896-10-31

**Authors:** 


### The Institutional Workshop.

### THE GUILD OF ST. LUKE.

### SERVICE AT ST. PAUL'S CATHEDRAL.

The thirty-second anniversary festival of the Guild of St. Luke, an association of medical practitioners and students who are communicants of the Church of England, took place on Wednesday, 21st inst., the annual service being held at St. Paul's Cathedral. As it had been suggested that some function for the medical profession, similar to that which now exists among the lawyers when Her Majesty's judges annually attend St. Paul's Cathedral in state, might well be inaugurated, it was felt by the guild that the occasion of the festival this year would be a suitable one for initiating such a ceremony. Accordingly invitations to the festival were issued to all the practitioners of medicine resident in London, those practitioners who are university graduates being asked to wear the gown and hood of their degree. The Lord Mayor consented to attend in state, and in the result an immense congregation assembled at the Cathedral.

The medical men who accepted the invitation of the guild, estimated at about 1,000, and the Lord Mayor's party were received on their arrival at the west entrance of the Cathedral, where a procession was formed. The choir (composed of over 200 members of the London Gregorian Association) and clergy led the procession, and following came the Lord Mayor and Sheriffs Ritchie and Rogers, attended by the Lord Mayor's Chaplain, the City Marshal, and the Sword and Mace Bearers. Then came the members of the guild in scarlet robes and academical hoods, and, finally, other members of the medical profession in hoods and gowns. The civic dignitaries took their seats in the choir, while the medical men had the whole of the space under the dome reserved for their use. Among those present were: Sir William Broadbent, Sir W. O. Priestley, Sir Henry Acland, Sir Dyce Duckworth, Dr. Theodore Williams, Dr. Oliver Codrington, Dr. W. S. Church, Dr. Bowles, Dr. Cotton (Ramsgate), Dr. W. M. Ord, Dr. Isambard Owen (Deputy Chancellor of the Welsh University), Mr. C. N. Macnamara (Vice-President of the Royal College of Surgeons), Dr. Roberts, Dr. Pavey, Mr. G. Cowell, Mrs. Scharlieb, M.D., Mrs. Barry, Dr. E. Symes Thompson (Provost of the Guild of St. Luke), Dr. S. Russell Wells and Mr. C. Devereux Marshall (hon. secretaries of the committee).

The evening service was intoned by Minor Canon Milman, and the lessons (Eccles. xxxviii., verses 1 to 15, and St. Luke xvi.) were read by the Rev. H. Arnott, F.R.C.S., and the Rev. Dr. Belcher, M.D. The service was made as special as possible: The first lesson, as our readers will see, was most apposite to a gathering of medical men; the second was taken from St. Luke, while the Special Collect for St. Luke's Day was not only read during the service, but was used by the preacher before commencing his sermon.

The late Archbishop of Canterbury had promised to preach the sermon on this occasion, but owing to his death, the Canon in residence (the Bishop of Stepney) undertook the duty.

The Bishop of Stepney, taking for his text Psalm lxix. 23, "Let the things that should have been for their wealth be unto them an occasion of falling," dwelt on the danger all classes of men and women were in of losing sight of the spiritual side of things through the close study of and familiarity with the material side. For instance, on the previous day he had spent some three hours in one of the largest of the London hospitals, speaking to the patients, and holding a service for a large number of convalescents. The whole thing—the neatness, the happiness, the quiet, the peace and repose of everything—was to him a matter of delight, but at the same time he felt that if he went through it all, as the workers in hospitals did, day after day, that delight would grow less and less, and he would become, as hospital workers were constantly struggling not to become—many of them most successfully—a merely useful machine. So, too, medical men knew what it was to become so immersed in the symptoms they were treating, so concentrated on that which was primary if by such powers as they possessed they were to bring back the physical frame of a patient to health and strength, that it did not occur to them that in the day of sickness the influence of the doctor for good could effect far more for the patient's spiritual benefit than all the medicines, or all his sermons could ever hope to effect. The young medical man, too, was trained to fix his attention upon the material. The one important thing to him was to master the secrets and laws of the material, not to wander off into speculations and dreams, but just to confine himself to the material and make the material his servant. It was right that this should be so. It must be so, for the enormous field to be covered was much more than enough to absorb his mind. He (the preacher) had played his little part in helping to develop in the last thirty years the wonderful Medical Schools that existed at Cambridge and other places, and he had seen things grow in marvellousness and complexity from what used to be a simple matter in the hands of three or four professors, had watched it become subdivided again and again, till at last people had come to realise that there was no part of nature so trivial or small that it might not be made the subject of the study of the very highest intellects, requiring laboratories, experimenting rooms, and machinery of the most costly description. With these surroundings he did not wonder that the young man became instinctively, as it were, a materialist. It could hardly be otherwise. But was that to continue to be so? Their business that evening was to think something about the harmony of religion and science. He was quite clear that religion could not do without science, and he had a very profound conviction that science could not do without religion. In the harmony of the two, each in its perfection and in its own sphere, he found the healing of this world and the building up of the next. The danger of the present day was lest scientific men should be lost in the mazes of materialism and not have the time, nor the inclination, nor the will, to look to the spiritual. He believed that if they had in the medical schools three or four right thinking young men—(for after all, if they could influence the young men they might leave the middle aged and old men to look after themselves)—men not less keen or clever than their keenest or cleverest companions—just the mere fact that, though they never gave it voice (indeed if they did give it voice they marred their opportunity), they were looking through the material to the marvellous spiritual world which lay beyond, would make them an influence which would be felt by all. Then let the mass of students, so very many of whom were men of the deepest religious instincts, join some guild (the Guild of St. Luke for instance) which had indirectly for its object the working for the harmony of religion and science, and he believed the danger to which he had alluded would be in a great measure overcome. In conclusion the Bishop said: This pulpit, this evening, is empty and silent—miserably empty and wofully silent. It is empty of the spirit that would have filled you all; silent of that marvellous voice that, once heard, you would never have forgotten—which would have said to you things that not all the anxieties of your profession would ever have been able to remove from your minds. It is miserably and wofully silent and empty, just occupied as a mere piece of Cathedral duty by the Canon of St, Paul's who happens at the time to be in residence. Do you need a more speaking message of how poor and weak and feeble a thing materialism is when you meet such a blow as we have met, and have to find what consolation you can, what strength you can, what hope you can—not one single one of which comes from any one material particle ever created on the face of the earth? It is into the world of the spirit that you are plunged when you try to get strength, comfort, and hope from that wonderful life and marvellously beautiful death. If the Archbishop had been here, his heart would have leapt with joy within him to see this enormous assembly, and to know the spirit that underlies it, not voiced by any of you, but henceforth to be voiced. His eye would have delighted to mark this pomp and circumstance—his eye that was so keen to mark and skilled to guide anything of the kind. In heart and body he would have revelled in all that is here and its enormous possibilities. He would have told you that when he wanted a man—a really fit man—to send as a medical missionary under conditions of great peril, he turned to the Guild of St. Luke and found him. He would have urged you to knit yourselves together, each in himself and with his companions, so that profound effect may be produced by the labour of love that has brought together such a gathering as I have never seen in this place, under circumstances that make the occasion absolutely and entirely unique. He would have urged you to do what you can to support and extend the comforting sense of the inalienable harmony between science and religion, and I pray God that that great and noble voice, if now silent through death, though silent may yet speak to every soul to whom I now speak.

At the close of the service a collection was made in aid of the Medical Missionary Fund of the Guild.

